# Lack of Association between the Tagging SNP A+930→G of SOCS3 and Type 2 Diabetes Mellitus: Meta-Analysis of Four Independent Study Populations

**DOI:** 10.1371/journal.pone.0003852

**Published:** 2008-12-04

**Authors:** Antje Fischer-Rosinsky, Eva Fisher, Peter Kovacs, Matthias Blüher, Matthias Möhlig, Andreas F. H. Pfeiffer, Heiner Boeing, Joachim Spranger

**Affiliations:** 1 Department of Endocrinology, Charité-Universitätsmedizin Berlin, Campus Benjamin Franklin, Diabetes and Nutrition, Berlin, Germany; 2 Department of Clinical Nutrition, German Institute of Human Nutrition Potsdam-Rehbrücke, Nuthetal, Germany; 3 Department of Epidemiology, German Institute of Human Nutrition Potsdam-Rehbrücke, Nuthetal, Germany; 4 Interdisciplinary Centre for Clinical Research, University of Leipzig, Leipzig, Germany; 5 Department of Internal Medicine III, University of Leipzig, Leipzig, Germany; University of Bremen, Germany

## Abstract

**Background:**

The suppressor of cytokine signalling 3 (SOCS3) provides a link between cytokine action and their negative consequences on insulin signalling. Thus SOCS3 is a potential candidate gene for type 2 diabetes (T2DM).

**Methodology/Principal Findings:**

Based on HapMap we identified the polymorphism A+930→G (rs4969168) as a haplotype tagging SNP (htSNP) sufficiently covering the genetic variation of the whole gene. We therefore examined the association between rs4969168 within SOCS3 and T2DM in three independent study populations; one prospective case-cohort study and two cross-sectional study populations. Due to the low frequency of individuals being homozygous for the polymorphism a dominant model of inheritance was assumed. The case-cohort study with 2,957 individuals (764 of them with incident T2DM) showed no effect of the polymorphism on diabetes risk (hazard ratio (95%CI): 0.86 (0.66–1.13); p = 0.3). Within the MeSyBePo-study population 325 subjects had T2DM from a total of 1,897 individuals, while the second cross-sectional cohort included 851 cases of T2DM within a total of 1653 subjects. According to the results in the prospective study, no association with T2DM was found (odds ratio (95%CI): 0.78 (0.54–1.12) for MesyBepo and 1.13 (0.90–1.42) for the Leipzig study population). There was also no association with metabolic subtraits such as insulin sensitivity (p = 0.7), insulin secretion (p = 0.8) or the hyperbolic relation of both, the disposition index (p = 0.7). In addition, no evidence for interaction with BMI or sex was found. We subsequently performed a meta-analysis, additionally including the publicly available data from the T2DM-subcohort of the WTCCC (n = 4,855). The overall odds ratio within that meta-analysis was 0.96 (0.88–1.06).

**Conclusions/Significance:**

There is no strong effect of the common genetic variation within the SOCS3 gene on the development of T2DM.

## Introduction

The genetic impact on type 2 diabetes mellitus (T2DM) is well known. However, due to various reasons, including considerable heterogeneity of the disease, the identification of susceptibility genes is difficult and most associations have not been replicated.

The suppressor of cytokine signalling 3 (SOCS3) provides a molecular link between cytokine action and insulin signalling [1]. In addition, SOCS3 has been shown to mediate a reduction of β-cell volume and modulates cytokine signalling in pancreatic β-cells [2].

Thus, from a functional perspective, SOCS3 appeared to be a convincing candidate gene with respect to T2DM. We investigated the only tagging SNP A+930→G (rs4969168, noncoding) of the gene [3] to examine its genetic impact on T2DM and parameters of the glucose metabolism in three independent study populations; one prospective case-cohort study and two cross-sectional study populations. A meta-analysis including publicly available data was also performed.

## Results

We here investigated a potential association between the tagging SNP A+930→G of the SOCS3 gene with T2DM or associated subtraits in three independent study populations.

The replication rate of genotyping was 99% and the genotype distribution were in Hardy Weinberg Equilibrium (χ^2^_EPIC_ = 3.66; χ^2^_MeSyBePo_ = 0.13; χ^2^_Leipzig_ = 0.18). In all subsequent calculations exclusively the dominant model was analysed due to the low frequency of homozygous carriers of the. Cox proportional hazard and logistic regression models adjusted for age, gender and BMI did not show any significant associations between the polymorphism and T2DM (see table 1A–C). The association between the polymorphism and validated indices estimating insulin sensitivity was also investigated within the MesyBepo study population. Comparably to the lack of association with diabetes, no relation to insulin sensitivity (p = 0.7), insulin secretion (p = 0.8) or Disposition Index was found (p = 0.7) (see table 1D). In addition, no interaction between the polymorphism with BMI or sex was found with respect to T2DM.

**Table 1:** Results of the tagging SNP A+930→G (genetic dominant model) for A) the Cox model for T2DM in EPIC, B) the logistic regression model in MeSyBePo, C) the logistic regression model in the Leipzig cohort and D) for the linear regression model of D1) ISI-insulin sensitivity, D2) AUC_Insulin_/AUC_Glucose_-insulin secretion, D3) DI-disposition index.

**A)**			
	**Genotype (n_subcohort_/n_external cases)_**	**Hazard Ratio (95%CI)**	**p-value**
	GG (1,835/563)	1 (reference)	
	GA+AA (399+32/118+10)	0.86 (0.66–1.13)	0.3
**B)**			
	**Genotype (n_non-case_/n_case)_**	**Odds Ratio (95%CI)**	**p-value**
	GG (1227/268)	1 (reference)	
	GA+AA (322+23/55+2)	0.78 (0.54–1.12)	0.8
**C)**			
	**Genotype (n_non-case_/n_case)_**	**Odds Ratio (95%CI)**	**p-value**
	GG (621/642)	1 (reference)	
	GA+AA (170+10/202+8)	1.25 (0.95–1.66)	0.1
**D)**			
	**Genotype**	**Mean (±SD)**	**p-value**
D1)	GG	0.079±0.027	0.7
	GA+AA	0.078±0.030	
D2)	GG	45.69±30.17	0.8
	GA+AA	46.02±30.09	
D3)	GG	3.53±1.81	0.7
	GA+AA	3.49±1.92	

All models were adjusted for age, gender and BMI, respectively.

**Figure 1:**
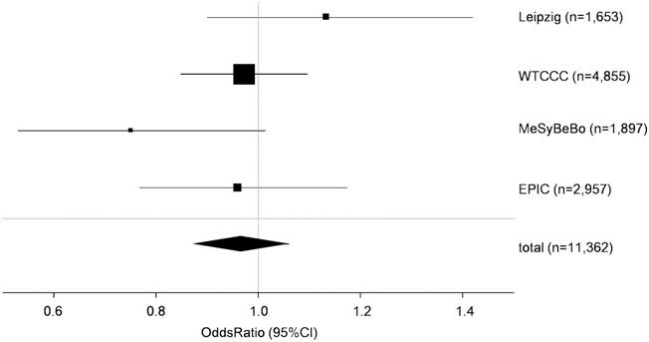
Forest blot presenting the meta-analysis of the study populations EPIC, MeSyBePo, Leipzig and the WTCCC. The size of each square is proportional to the study's weight within the meta-analysis. The overall effect estimate is plotted as a diamond. The lateral points of that diamond indicate the 95%-CI of this estimate.

We also performed a meta-analysis using the here genotyped three study popualtions and publicly available data from the WTCCC, resulting in a total 11,335 individuals. Crude odds ratios were calculated for this meta-analysis due to limited access to individualized information within the publicly available data. In addition, the different study designs need to be considered for interpretation of the meta-analysis. Crude OR was 0.95 (95%CI 0.77–1.17) for the EPIC-Potsdam cohort, 0.73 (95%CI 0.53–1.01) for the MeSyBePo study population, 1.13 (95%CI 0.90–1.42) for the population from the region of Leipzig and 0.96 (95%CI 0.85–1.10) for the T2DM-subcohort in the WTCCC. Meta-analysis revealed a total odds ratio of 0.96 (95%CI 0.88–1.06) (Figure 1). Genotype frequencies of all study populations are shown in table 2. Power calculations revealed that the meta-analysis provided 80% power to detect a 12% risk modification.

**Table 2:** Genotype frequency of the tagging SNP A+930→G in the three study populations included in the meta-analysis.

	genotype	study population (n %)				total
		EPIC	MeSyBePo	WTCCC	Leipzig	
cases	AA	11 (1.4%)	2 (0.6%)	38 (2.0%)	8 (0.9%)	59 (1.5%)
	AG	129 (16.8%)	56 (17.2%)	491 (25.6%)	202 (23.7%)	871 (22.7%)
	GG	624 (81.8%)	267 (82,2%)	1,393 (72.4%)	642 (75.4%)	2,907 (75.8%)
	Total	764	325	1,922	852	3,837
controls	AA	31 (1.4%)	22 (1.4%)	71 (2.4%)	10 (1.3%)	134 (1.8%)
	AG	388 (17.7%)	323 (20.6%)	758 (25.8%)	170 (21.2%)	1,638 (21.9%)
	GG	1,774 (80.9%)	1,227 (78.0%)	2,104 (71.8%)	621 (77.5%)	5,726 (76.3%)
	Total	2,193	1,572	2,933	801	7,498

## Discussion

From a functional perspective, SOCS3 is a convincing candidate gene for genetic association studies investigating susceptibility for T2DM. This study examined a variant in the 3′ UTR of SOCS3 for association with T2DM and related traits. This variant covers the genetic variation within SOCS3 according to HapMap data, thus being the only haplotype tagging SNP within this gene [3]. The samples included a total of 1897 individuals from the cross-sectional MeSyBePo study, 1653 individuals from a cross-sectional study from the region of Leipzig and 2957 individuals from the prospective EPIC-Potsdam study. Surrogate measures of insulin secretion and sensitivity were obtained in non-diabetic MeSyBePo participants. A meta-analysis of the here genotyped data and publicly available data from the WTCCC was additionally performed. This meta-analysis had an 80% power to detect effect sizes of 12% given the frequency of the here investigated polymorphism. All results are negative for association with T2DM or related traits.

Some shortcomings of this study should be mentioned. All high-density genome-wide association studies have not found effects within the region of SOCS3. Given the existing data the study may be underpowered to demonstrate small associations, which are not entirely unlikely given the existing results of genome-wide association studies. Aiming to investigate those smaller effects the here presented data may be valuable for future meta-analyses in combination with additional future available data. The existing data of genome-wide scans did not investigate potential interaction with environmental factors such as BMI or sex. Those interactions are unlikely to exist given our data. However, the analysis of interactions further reduces the power of studies making future meta-analyses also desirable.

In conclusion, we were unable to detect an effect of the one tagging SNP A+930→G of the SOCS3-gene on T2DM or associated subtraits. Although a substantial effect of this SNP can be excluded, small risk modifications may still exist, which should be investigated in future meta-analyses.

## Materials and Methods

Details of recruitment and phenotyping (Case/Control: age 59.57/50.63 years; ♀ 181/1088; ♂ 144/484; BMI 32.24/28.67) of the MeSyBePo-study were published recently [4]. In all participants of MeSyBePo a 75 g oral glucose tolerance test (OGTT) with insulin measurements was performed. 325 participants with T2DM were compared to 1,572 non-affected individuals. The association between the polymorphism and the Disposition Index was investigated only in individuals with NGT, IFG or IGT, since accepted markers of insulin sensitivity and secretion have been shown to be unreliable in patients with type 2 diabetes mellitus, especially in patients with anti-diabetic treatment. An euglycemic hyperinsulinemic clamp was performed in a subset of 56 healthy controls. In these participants, the Insulin Sensitivity Index (ISI) according to Stumvoll (ISI was calculated as: 0.157–4.576 10^−5^ Ins_120_– 0.00519 Gluc_90_ – 0.000299 Ins_0_) correlated best to the M-value (r = 0.591; p<0.001) of the clamps [5]. Therefore ISI was subsequently used to estimate insulin sensitivity in the total cohort. As the characteristics of our study population were basically comparable to those of Stumvolls population, the ratio of AUC_Insulin_/AUC_Glucose_, which performed best in Stumvolls study compared to a hyperglycemic clamp, was used to estimate insulin secretion. Correspondingly the disposition index (DI) was calculated as the product of ISI and AUC_Insulin_/AUC_Glucose_ [5], [6].

A second cross-sectional study population was from the region of Leipzig. DNA from 852 patients with T2DM (♀ 408, ♂444) and 801 non-diabetic subjects (♀ 555, ♂ 246) recruited at the University Hospital in Leipzig, Germany were available for the present study. The non-diabetic subjects had mean age 49±14 years and mean BMI 28.6±5.6 kg/m^2^, and patients with T2DM had mean age 64±11 years and mean BMI 29.6±5.2 kg/m^2^ (arithmetic means±SD). In addition, OGTT was performed in all non-diabetic subjects. Since impaired glucose tolerance is a T2DM predicting factor, only subjects with normal glucose tolerance (N = 633) were included as healthy controls in the T2D case-control study.

Association with incident T2DM was assessed in a nested case–cohort study within the EPIC-Potsdam- (European Prospective Investigation into Cancer and Nutrition) study, a prospective cohort involving 27,548 Caucasian volunteers mainly aged 35–65 years from the general population [7]. The case-cohort has been described in detail before [8]. 2,266 individuals were randomly selected for a subcohort and 764 incident cases were included in the present analysis. Because the subcohort is representative of the entire cohort at baseline, 73 incident T2DM were included in the subcohort and 691 incident cases were identified in the remaining cohort, the latter classified as ‘external’ cases (external cases/subcohort subjects: age 54.7/49.5 years; ♀ 280/1,401, ♂ 411/865; BMI 30.4/26.0, mean follow up: 7 years). Both studies have been approved by the local ethic authorities, namely the EPIC-study was approved by the ethic committee of Brandenburg and MeSyBePo was approved by the ethic committee of the University of Potsdam and the Charité-Universitätsmedizin Berlin. All study participants gave written informed consent to the studies, respectively.

Genotyping was performed by TaqMan®-technology (HT7900 System; ABI, Foster City, CA, USA). Details of genotyping will be given by the authors upon request. The polymorphism was chosen by the tag SNP Picker on the International HapMap Project web side with an r^2^-cut off of 1 and a minor allel frequency cut off of 0.1 [3].

Finally publicly available data of a sub-cohort of the WTCCC (Welcome Trust Case Control Consortium) with genotyped data of the SOCS3 polymorphism were used including a total of 4,355 persons (n_case_ = 1,.422; n_control_ = 2,933).

Data were analyzed using SPSS (SPSS Inc., Chicago, IL, USA, version 12.0) and SAS (SAS Institute, Cary, NC, USA, version 9.1). General linear model was calculated to analyse the effects of the polymorphism on continuous variables after adjustment for confounders (age, sex, BMI). Hazard ratios were calculated using Cox proportional hazard regression analysis modified according to the Barlow method in EPIC-Potsdam. Unconditional logistic regression analysis was performed to estimate prevalent odds ratios in MeSyBePo. Multiplicative interaction terms between the genotype and age, sex or BMI, respectively, were used to analyze potential interactions of these variables and disease risk. A two-tailed alpha-error below 5% was considered to be significant.

Power analysis was performed using Quanto [9]. We assumed an unmatched case-control design (EPIC 1∶3.4, MeSyBePo 1∶3.7, Leipzig 1∶0.94), a population risk of 0.06 for T2DM and considered a two-sided p-value of 0.05. Meta-analysis has been performed using the Mantel-Haenszel method, which assumes a fixed effect and combines studies according to the weight given to each study. All analyses were performed using a dominant genetic model, due to the low frequency of homocygote mutant carriers.
